# Clinicians Primarily Practicing in Nursing Homes and Care Quality for Residents with Dementia

**DOI:** 10.1093/geroni/igaf122.2686

**Published:** 2025-12-31

**Authors:** Hyunkyung Yun, Mark Unruh, Yongkang Zhang, Yuting Qian, Hye-Young Jung

**Affiliations:** Brown University, Providence, Rhode Island, United States; Joan and Sanford I. Weill College of Medicine, Cornell University, New York, New York, United States; Weill Cornell Medicine, New York, New York, United States; Yale School of Public Health, New Haven, Connecticut, United States; Joan and Sanford I. Weill College of Medicine, Cornell University, New York, New York, United States

## Abstract

Physicians and advanced practitioners (APs) who primarily practice in nursing homes (NHs), often called “SNFists,” provide care in more than 50% of U.S. NHs. Little is known about SNFists’ impact on care quality for long-stay NH residents with dementia. We estimated the association between receipt of care from a SNFist and care quality for these residents using claims for a nationwide sample of Medicare beneficiaries, 2013–2019. The intervention of interest was resident attribution to a SNFist based on a plurality of evaluation and management visits. SNFists included generalist physicians and APs. Estimation was based on a machine learning approach incorporating a doubly-robust procedure using a generalized estimating equation with inverse probability treatment weighting. We also conducted stratified analyses for physicians and APs, and a sensitivity analysis that included physicians of any specialty, and APs. Among the 417,378 residents in our sample, 58% (242,540) were attributed to SNFists. In adjusted analyses, attribution to a SNFist vs. a non-SNFist was associated with 7% lower odds of an ambulatory care sensitive (ACS) hospitalization (OR: 0.93; 95% CI, 0.90-0.96) and 7% lower odds of an ACS ED visit (OR: 0.93; 95% CI, 0.90-0.96). In stratified analyses, attribution to a physician SNFist vs. non-SNFist physician was associated with 13% lower odds (95% CI, 0.83-0.90) and 7% lower odds (95% CI, 0.88-0.97) of an ACS hospitalization or ED visit, respectively; AP-SNFist comparisons were not statistically significant. Results of the sensitivity analysis were consistent with primary results. SNFists may enhance care quality for residents with dementia.

